# The Ethical Obligation for Research During Public Health Emergencies: Insights From the COVID-19 Pandemic

**DOI:** 10.1007/s11019-023-10184-6

**Published:** 2023-12-28

**Authors:** Mariana Barosa, Euzebiusz Jamrozik, Vinay Prasad

**Affiliations:** 1 Nova Medical School, Nova University of Lisbon, Lisbon, Portugal; 2 Science and Technologies Studies (MSc student), University College London, London, UK; 3 Ethox and Pandemic Sciences Institute, University of Oxford, Oxford, UK; 4 Royal Melbourne Hospital Department of Medicine, University of Melbourne, Melbourne, Australia; 5 Monash Bioethics Centre, Monash University, Melbourne, Australia; 6 University of California, San Francisco, 550 16th St, San Francisco, CA 94158 USA

**Keywords:** Medical research ethics, Public health research ethics, Non-pharmaceutical interventions, Public health emergency, Pandemic

## Abstract

In times of crises, public health leaders may claim that trials of public health interventions are unethical. One reason for this claim can be that *equipoise*—i.e. a situation of uncertainty and/or disagreement among experts about the evidence regarding an intervention—has been disturbed by a change of collective expert views. Some might claim that equipoise is disturbed if the majority of experts believe that emergency public health interventions are likely to be more beneficial than harmful. However, such beliefs are not always justified: where high quality research has not been conducted, there is often considerable residual uncertainty about whether interventions offer net benefits. In this essay we argue that high-quality research, namely by means of well-designed randomized trials, is ethically obligatory before, during, and after implementing policies in public health emergencies (PHEs). We contend that this standard applies to both pharmaceutical and non-pharmaceutical interventions, and we elaborate an account of equipoise that captures key features of debates in the recent pandemic. We build our case by analyzing research strategies employed during the COVID-19 pandemic regarding drugs, vaccines, and non-pharmaceutical interventions; and by providing responses to possible objections. Finally, we propose a public health policy reform: whenever a policy implemented during a PHE is not grounded in high-quality evidence that expected benefits outweigh harms, there should be a planned approach to generate high-quality evidence, with review of emerging data at preset time points. These preset timepoints guarantee that policymakers pause to review emerging evidence and consider ceasing ineffective or even harmful policies, thereby improving transparency and accountability, as well as permitting the redirection of resources to more effective or beneficial interventions.

## Introduction

**Fig. 1:**
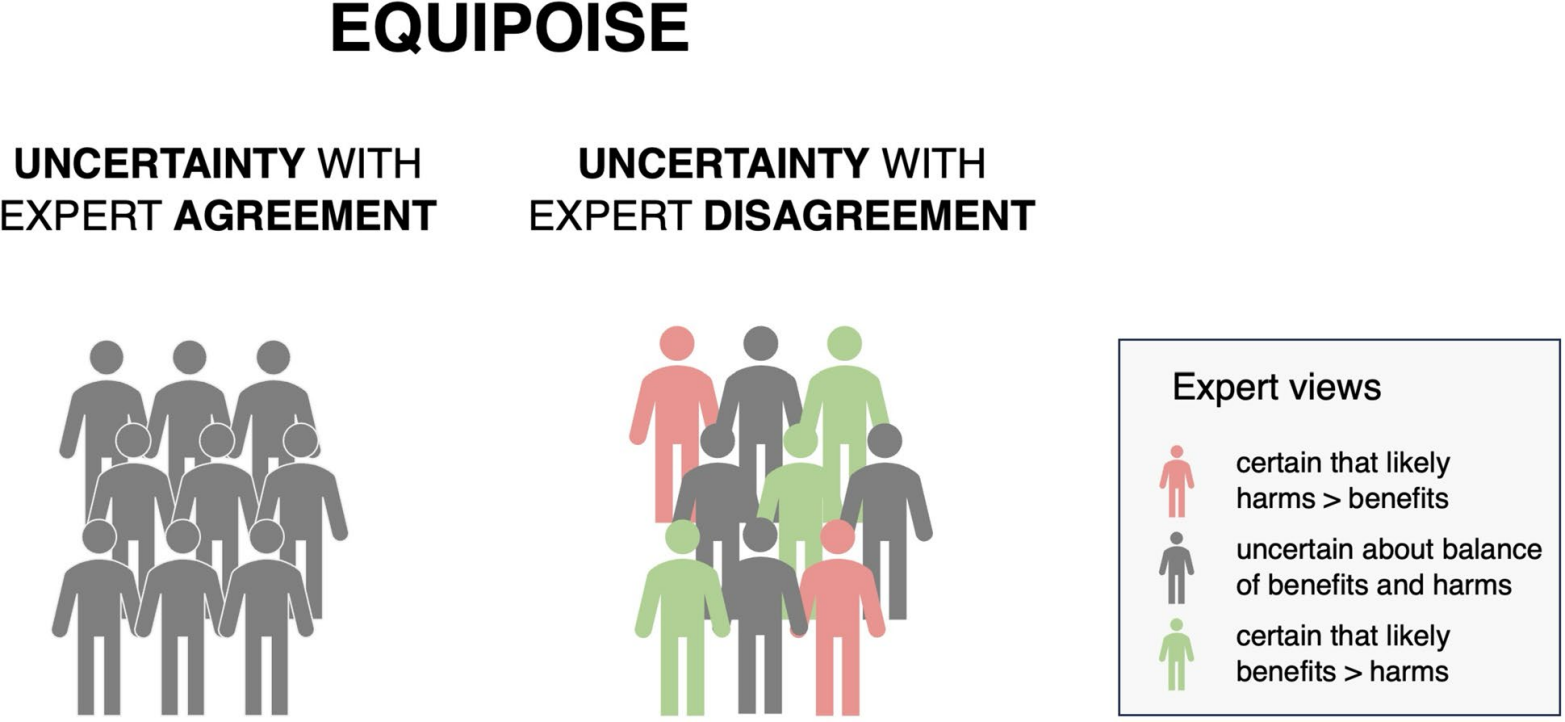
Equipoise in situations of uncertainty and/or disagreement

The concept of equipoise has long been used in research ethics to guide decisions in situations of uncertainty about trials of interventions. Yet there has been relatively little attention in public health ethics to how public health policymakers ought to make decisions about interventions whose benefits and harms are uncertain. In research ethics, equipoise denotes a situation of collective uncertainty and/or disagreement among experts about the evidence regarding an intervention (London 2020, 81). In situations of equipoise about an intervention, it is widely considered ethical to conduct trials of the intervention in order to change expert views about the intervention and thus disturb equipoise either for or against its use. In situations of equpoise, experts may hold different views about an intervention (Fig. 1), and disagreements may become more acute during public health emergencies. During emergencies, scientists may feel pressured to present the collective state of expert views as a consensus in order to promote public support of interventions imposed by public health agencies even where significant doubts or disagreements persist - e.g., where  a significant and well-informed minority of experts disagrees with a majority view (Intemann & de Melo-Martin 2023). In such contexts, public health leaders may claim that equipoise has been disturbed (by the supposed consensus) and that further trials would be unethical despite considerable residual uncertainty. This illustrates a link between ethically acceptable research and policy: the implementation of policy interventions (and/or the perceived disturbance of equipoise) sometimes forecloses opportunities for continued research.

Like all public health emergencies, the COVID-19 pandemic has been associated with significant uncertainty, from basic facts about the virus to the best policy responses—what interventions policymakers ought to choose in order to minimize mortality and morbidity with the least countervailing harmful, disruptive, and unanticipated effects. To date, the appropriate role of non-pharmaceutical interventions (NPIs)^1^ including “lockdowns”; business, school, and playground closures; community masking; distancing; testing; screening; quarantining; and other policies remains hotly debated (Dahlquist and Kugelberg 2023).

Good science should aim to resolve uncertainty. In part this is because ethical policy responses require reliable evidence that expected benefits (i.e., real-world effectiveness) outweigh expected harms (Jamrozik 2022). Major policy decisions should arguably be guided by more than observational data (or mere association between a policy and an outcome). From a methodological perspective, randomized controlled trials (RCTs) are typically the best way to establish causal claims regarding “efficacy” and, in some situations, the “effectiveness” of interventions (Broadbent 2013, 262–270; Howick 2011, ch.5). Well-designed RCTs arguably reach more reliable causal conclusions than observational studies^2^ and generally require that fewer people are subject to less effective therapy/intervention, may produce results faster, and often cost less than implementing an intervention in practice before making assessments of effectiveness (Prasad 2021). Yet it is often claimed that randomized trials are unethical, unfeasible, or unnecessary, particularly during public health emergencies (PHEs) (Brouqui and Raoult 2020; Adebamowo et al. 2014). The pandemic arguably exposed a decline of evidence-based policy paradigms insofar as observational evidence was often privileged over evidence from RCTs (Ferreira et al. 2021; Haber et al. 2022).

Here, we argue that in public health crises it is not merely ethically permissible, but there is an ethical obligation to conduct high-quality randomized studies in order to reduce uncertainty, thereby disturbing equipoise by the accumulation of rigorous scientific evidence (rather than by a change in expert views based on less rigorous evidence or other factors). We argue that rigorous studies are therefore ethically obligatory before, during, and after implementing policies.^3^ We appeal to equipoise because it captures aspects of uncertainty and disagreement among experts that were key features of debates during recent public health emergencies. Although outside the scope of the current paper, other ethical justifications for research, for example those that appeal to social or scientific value, will also require an account of expert consensus (e.g., regarding the social value of evidence generated by research) and may thus be informed by our analysis of expert consensus in the context of equipoise. Moreover, other authors have argued that proper appeals to equipoise entail considerations of social value (London et al. 2023).

This essay is divided into six main sections. First, we provide a theoretical background to why high-quality research is ethically obligatory in PHEs. Second, we argue that there is no relevant ethical difference between NPIs and pharmaceutical interventions. Third, we explore options for research trials before, during and after policy implementation in PHEs. Fourth, we provide case studies of evidence generation regarding COVID-19 drugs, vaccines, and non-pharmaceutical interventions. Fifth, we consider objections and responses. Sixth, we propose a key reform for evidence-based policies in public health emergencies: whenever a policy is not based on prior high-quality evidence, there should be a planned approach to generate high-quality evidence, with review of emerging data at preset time points. Finally, we present our conclusions.

## Section 1: Why high-quality research is ethically obligatory in public health emergencies

### Equipoise and public health emergencies

Equipoise denotes a situation of uncertainty and/or disagreement among experts about the evidence regarding an experimental intervention (Fig. 1) (London 2020). It is a widely endorsed ethical standard for both clinical and public health research because it helps to determine: (1) when trials are ethical (and, by extension, when further trials would be unethical) and (2) when there is sufficient evidence that implementing an intervention as policy would be expected to produce benefits that outweigh risks. According to this standard, conducting a research trial is ethical if there is equipoise about whether an intervention is better than an alternative (including a placebo, or no intervention), in terms of having a superior balance of benefits over harms and other costs.

In situations of uncertainty, there may be different views about how to proceed in the face of disagreements. There may be political pressure for scientists to present the collective state of expert views as one of consensus, resulting in a “curated consensus” rather than a spontaneous consensus based on independent assessments of available data (Godfrey-Smith 2003, 81). A claim that equipoise is disturbed, e.g., that the consensus view of experts is that the expected benefits of an intervention outweigh its risks, may entail (1) the ethical acceptability of implementing the intervention and/or (2) that further research on the intervention (at least in similar contexts to existing research) is unethical (London 2009). However, even in the presence of majority expert consensus, significant doubts or disagreements may persist among a significant well-informed minority of experts. This is why some analyses of equipoise suggest that, in the presence of such disagreements, ongoing research may be ethical, including alongside policy implementation (London et al. 2018). While there may be challenges in characterizing expert community uncertainty and operationalizing the concept of equipoise (London 2020), insofar as expert community views can be characterized, this may help to prioritize among research questions (or, in other words, to decide which uncertainties should be addressed first). For example, a well-designed randomized trial might be more likely to make a meaningful difference to overall consensus in situations where expert views are “skewed” in that only a small minority of experts regard a novel intervention as likely to be superior to standard practice (and where both the minority and a substantial fraction of the majority would be willing to revise their views in light of the results of the trial) (London et al. 2023)—and such situations may be common in public health emergencies. In contrast, when expert views are spread more evenly, other types of evidence generation (including observational data or mechanistic studies) may help to build some degree of consensus one way or the other and prepare the ground for more definitive trials (London et al. 2023).

At the outset of a PHE, it will often be uncertain whether some drugs, vaccines or NPIs will have expected benefits that outweigh their harms and costs. If an uncertain intervention has never been trialed for the problem at hand, all expert views will have an arguably fragile epistemic basis but there may nevertheless be disagreements among experts with, as above, a minority favoring the novel intervention while others remain uncertain or prefer standard practice. The only way to settle expert disagreements and reduce uncertainty is to conduct *high-quality research* that will convince neutral or skeptical experts to change their views. Hereafter we use the term *high-quality research* when referring to research that offers high-quality evidence supporting causal claims about expected benefits and harms of interventions.

### An ethical case in favor of high-quality research during public health emergencies

#### A. Disturbing equipoise

Equipoise in the context of PHEs can be perceived as problematic because PHEs demand urgent action. Uncertainty and/or disagreement among experts might be perceived to undermine such action. Nevertheless, research is arguably needed to provide high-quality data in order to inform expert judgements that implementing any given intervention is ethically acceptable (i.e., that there is adequate certainty of net benefit) (London 2019). What counts as "adequate certainty" may vary, but in any case our best attempts to attain it come from research methodologies that can best support causal conclusions and thereby convince a larger fraction of experts, especially those with the highest epistemic standards, to change their views. This is why, for example, regulatory agencies typically demand high-quality experimental data *before* the approval of pharmaceutical interventions. At the outset of PHEs, high levels of uncertainty about risk–benefit ratios means that the probability of causing net harm can be just as high as that of causing net benefit. Thus, there is an ethical case in favor of conducting high-quality research with the goal of disturbing equipoise.

#### B. Failing to disturb equipoise

Failure to disturb equipoise entails a significant probability that certain interventions are promoted on fragile epistemic bases and/or that expert disagreements become more marked as groups with different views become segregated from one another (London et al. 2023). Interventions that are implemented despite uncertainty about their effects can turn out to be ineffective, have a less favorable risk–benefit ratio than alternatives, or even be harmful. Failing to acknowledge these outcomes can lead to resource wasting and, worse, net harm for societies. Most public health interventions during PHEs require significant public resources from a finite pool, and societies expect governments to manage resources in an informed manner, such that the promotion of ineffective or even harmful policies is minimized. Of course, a single RCT may not always be definitive, and even well conducted RCTs can be contradicted by later ones (Prasad et al. 2013). Good policymakers always must therefore manage resources and make decisions balancing relative uncertainties given the available evidence and any changes in relevant circumstances. Yet, it is undesirable to allow continued uncertainty on issues of large consequence: the goal should therefore be to reduce uncertainty (eg. by conducting research to disturb equipoise) whenever possible. Among other things, this permits efficient re-direction of resources to more beneficial and/or less harmful interventions.

#### C. Preparedness for future PHEs

Future PHEs, particularly epidemics, are inevitable. Experience has shown that research plays a key role in both emergency preparedness and response, including via improved understanding of epidemic pathogens, the development of new interventions, and assessment of the effectiveness of responses (InterAcademy Partnership, Academy of Medical Sciences and Medical Research Council 2019; Eyal 2022). It is often the case that past epidemics and inter-epidemic research provide useful evidence for the management of a new epidemic, more so if it is embedded within a wider scientific capacity building agenda. For instance, knowledge about coronaviruses was useful to understand the basics of SARS-Cov-2, and research on masking during previous respiratory virus epidemics (eg. influenza) was useful, to some degree, to recommend masking to healthcare professionals (but not to members of the public) at the outset of the pandemic (World Health Organization 2020). If high-quality evidence exists, particularly regarding interventions, policymakers are better equipped to rapidly implement new policies with greater certainty of net benefit (Table 1).

**Table 1:** Examples of pragmatic study designs and potential applications to NPIs, with examples from the COVID-19 pandemic

	Design elements	Potential application	COVID-19 pandemic studies	Goals/advantages
**Pragmatic trial design** (can be mixed)				
SMART (Sequential, Multiple-Assignment Randomized Trial)	Pre-specified time points ± Clusters ± Stepped-wedge	Community re-openings and de-intensification of measures (eg. schools, restaurants) Community masking Prevention measures (social distancing, hand-washing, testing, etc.) Clinic-based telemedicine	(none found)	Adjustment to policy uptake Tailoring of interventions and sequencing Participation encouragement
Stepped-wedge^a^	Staggered roll-out Pre-specified time points ± Clusters ± Randomization ± SMART	Vaccine roll-outs Community re-openings (eg. schools, public spaces) Monitoring program	Pulse oximetry monitoring (retrospective)^1^ Quality improvement^2^	Assess effects of time Overcome logistic of financial constraints All participants have opportunity of treatment
Randomized preference^b^	Partial or full randomization ± Clusters	Contact tracing program incentives to quarantine	(none found)	Determine adherence to recommendations
Cluster RCT	Clusters of individuals as randomization units	Stay-at-home Information and education Masks and behavior change Testing strategy School closure and opening	Facebook ads to encourage stay-at-home^3^; Bangladesh masking^4^; Nursing home telemonitoring (ongoing)^5^; Best practices in care homes (ongoing)^6^; Testing strategy (ongoing)^7^; School opening (withdrawn)^8^	Practical feasibility of clusters Consent facilitation
**Pragmatic re-formulations of observational studies**^d^ (considerable limitations)	Combination of stepped-wedge and SMART			
Instrumental variable analyses		Social distancing	Mobility restrictions^9^	Estimate the local average intervention effect Address omitted bias^e^
Regression discontinuity	± Prospective	Masking	Children masking in schools^10^	
Natural experiment	Difference in differences	Stay-at-home Masking Social distancing School closures	Curfews^11^ N95 mask vs. surgical mask in Germany^12^ Hard vs soft lockdown^13^ Effect on young mental health in Germany^14^	

^a^Can be applied in a retrospective observational study

^b^Choices about different options (preferences)

^c^National ethical committee disapproval

^d^Not as reliable as experimental studies, but these are more robust methods to control for unobserved confounding than standard observational studies

^d^Still limited in terms of overall inferences

NPI: non-pharmaceutical intervention; RCT: randomized controlled trial

Adapted from Digitale et al. (2021)

^1^Beaney et al. (2022)

^2^Zhou et al. (2021)

^3^Breza et al. (2021)

^4^Abaluck et al. (2022)

^5^Calo et al. (2022)

^6^Levison et al. (2023)

^7^Hayes et al. (2022)

^8^Fretheim et al. (2020)

^9^Fakir and Bharati (2021), Brzezinski et al. (2020)

^10^Coma et al. (2023)

^11^deHaas et al. (2022)

^12^Miller (2022)

^13^Butterworth et al. (2022)

^14^Felfe et al. (2023)

## Section 2: The same principles apply to pharmaceutical and non-pharmaceutical interventions

Rigorous trials are required before drugs are approved, and the possibility of harm is widely acknowledged and usually feared. More than 2500 controlled trials have been registered for pharmaceutical interventions for COVID-19 (McCartney 2020). The value of such trials rests on their promise to produce reliable and generalizable evidence of net benefit. In principle, the same standards apply to NPIs (e.g., masks, school closures, vaccine policies, etc.) (Høeg and Prasad 2023). Yet, during the COVID-19 pandemic, there have been inconsistent approaches to pharmaceutical and non-pharmaceutical interventions; NPIs were often widely adopted without prior (or ongoing) testing (Høeg and Prasad 2023). Table 2 compares attempts of randomized investigation (i.e., registered trials) of select pharmaceutical and non-pharmaceutical interventions for COVID-19.

**Table 2:** Randomized trials of select pharmaceutical and non-pharmaceutical interventions against COVID-19^a,b^

**Pharmaceutical interventions**	RCTs	Ever mandated?^c^
Hydroxychloroquine	⊕⊕⊕⊕⊕⊕⊕⊕⊕⊕	No
Remdesivir	⊕⊕⊕⊕⊕⊕	
Tocilizumab	⊕⊕⊕⊕⊕	
**Non-Pharmaceutical interventions**		
Face masks	⊕⊕	Yes
School closure/opening	0^c^/⊕(1 trial^d^)	
Testing and screening strategies	⊕	
⊕: 1–5		
⊕⊕: 6–10	⊕⊕⊕⊕⊕: 21–25	⊕⊕⊕⊕⊕⊕⊕⊕: 36–40
⊕⊕⊕: 11–15	⊕⊕⊕⊕⊕ ⊕ : 26–30	⊕⊕⊕⊕⊕⊕⊕⊕⊕: 41–45
⊕⊕⊕⊕: 16–20	⊕⊕⊕⊕⊕⊕⊕: 31–35	⊕⊕⊕⊕⊕⊕⊕⊕⊕⊕: ≥ 45

^a^We retrieved information about randomized trials on non-pharmaceutical interventions from the scoping review by Hirt et al. (2022). The authors looked for trials until 17 August 2021, so we conducted a similar search on ClinicalTrials.gov and the WHO International Clinical Trials Registry Platform in order to compare similar time periods. We excluded trials testing the selected intervention in combination with another intervention

^b^Idea quoted with permission from Høeg and Prasad (2023)

^c^As of May 2023, there are no RCTs on school closures (Hume et al. 2023)

^d^Withdrawn; no reason provided in registry (Fretheim 2020)

RCT: randomized clinical trial

Like drugs and vaccines, it will often be the case for NPIs that (1) there are potential harms, including large economic costs (Lally 2022), as well as potential benefits; (2) there is an uncertain balance of benefits, harms, and costs prior to rigorous research; (3) they may be partly subsidized by public or shared resources; and (4) using unproven potentially ineffective NPIs may distract attention and resources from other more promising interventions. Additionally, (5) in contrast to drugs, NPIs often aim at whole populations, thus harms can even be greater (Bardosh et al. 2022a). These are all reasons why NPI research is arguably ethically acceptable and sometimes ethically obligatory (Bain et al. 2022).

One salient difference is that NPIs might be considered different *kinds* of interventions as compared with drugs or vaccines. Some NPIs are products (e.g., masks, air purifiers, etc.) while some are not (e.g., curfew, school closure, etc.). Even those that are products are typically subject to less rigorous regulation than pharmaceutical products and may not need to be produced in specialized regulated facilities. Arguably this means that proof of safety and net benefit is all the more important, given the lack of other safeguards.

The fact that NPIs are used in complex behavioral and social systems is sometimes a reason why experimental studies of NPIs are deemed unfeasible and/or not externally valid (i.e. because results in one setting may not be considered generalizable to other settings). However, the appreciable innovation in trial design has shown that there are methods that largely eliminate those concerns. New trial designs include cluster RCTs, multi-arm platform trials and adaptive trials. Many of these are also pragmatic studies, that is, randomized or quasi-experimental studies whose goal is to generate evidence for implementation of an intervention into real-world practice (Digitale et al. 2021). In Table 1 we show several examples of pragmatic study designs and potential applications to NPIs, with examples from the COVID-19 pandemic. Although these designs may require additional preparation, they are critical if we want to find out the most impactful interventions and should arguably be planned as early as possible. A common concern is that given the number of interventions, the range of potential study populations and the number of settings in the course of a pandemic, the number of possible combinations would reach the tens or hundreds of thousands (Kimmelman and London 2015). But platform trials, for example, can simplify trial logistics by evaluating multiple interventions in a single trial, and there is evidence that they can drastically reduce cost and efforts (Park et al. 2022).

## Section 3: Research before, during, and after policy implementation

### Research before policy implementation

High-quality research before policymaking in PHEs may be facilitated in different ways. On the one hand, high-quality research can be conducted between PHEs. For example, pre-clinical and clinical research on pathogens of pandemic potential (or related pathogens) can inform policymaking during a new PHE. On the other hand, preparation for conducting research during emergencies can also begin in inter-epidemic periods. This can be done by developing pre-approved generic trial designs that can be rapidly updated at the start of an emergency with the specifics of the pathogen and the NPI, or by building institutional support for large scale policy trials in PHE. Furthermore, trials designs can be modified in response to community preference. For example, trials of Ebola drugs excluded the use of placebo (instead comparing multiple experimental drugs) (Mulangu et al. 2019) and an Ebola vaccine trial used a stepped wedge rather than standard randomized design. Some of these changes may reduce the utility of results to some degree, but still enable more reliable causal inferences than observational data (London et al. 2018).

### Research during and after policy implementation

As a PHE unfolds, large amounts of data on the pathogen are generated and some interventions are tested in more or less rigorous trials yet, despite these knowledge gains, (i) some questions are still left unanswered despite the emergence of new data and (ii) baseline conditions change (e.g., more people become immune via vaccination or prior infection). Initial studies suggesting that expected benefits of an intervention outweigh risks often still contain residual uncertainty, and data collection after implementation can alter assessment of risks and benefits (for example, by identifying groups where the benefits or harms of an intervention may be particularly high). In Table 3 we show different strategies that promote research during and after policy implementation and their ethical relevance.

**Table 3:** Ethical relevance of different strategies that promote research during and after policy implementation.

Strategy	Ethical relevance
Extension of initial trials	It may be ethical to continue a trial, for example of a vaccine, even once it becomes clear that the vaccine offers benefits compared to placebo. For instance, delaying unblinding of participants—provided that this would not result in unreasonable risks to participants—in order to collect additional data (e.g., on post-vaccination infections). At a minimum, blinding could have been preserved for lower risk populations within a trial (e.g. for COVID19 those aged 18–28, and not 75–85) Similarly, where experts disagree about whether initial trials provide enough data to reduce uncertainty to a sufficient degree (i.e., sufficient to justify implementing an intervention)^b^, there may be situations where a reasonable minority of experts consider that further randomized research data should be gathered even while an intervention is being implemented
Implementation trials	It will often be essential from a practical perspective, but also ethical, to implement an intervention in a stepwise fashion in different segments of a population over time. Among other trial designs, this can allow for stepped-wedge randomized trials to collect additional data during implementation of an intervention presumed to be net beneficial (based on earlier standard trials)
Repeating trials in new populations	Additionally, where initial trials may not be generalizable to other population groups (not included in prior trials), it is ethical to start using an intervention in some population groups while gathering more randomized data in others. For example, the recommendation of additional doses of the COVID-19 vaccine for older groups without robust evidence may be reasonable, but not so for young patients, to whom net benefits are less certain
Changing policy in light of new evidence	The implementation of interventions without robust scientific evidence may be ethically acceptable during the initial period of a PHE. Even though this is a period of societal adjustment on many levels, public health experts should recognize the importance of generating high-quality evidence as soon as possible in order to provide evidence to continue (or discontinue) specific interventions. As time passes, if no experimental high-quality research is conducted, the evidence supporting these interventions will still be weak, uncertainty about the risk–benefit ratio will persist
Post-implementation data collection	Some forms of non-experimental post-implementation research, such as the collection of adverse events and effectiveness data, are essential to evaluate the balance of benefits, harms, and costs in practice, and thereby the ethical acceptability of continuing to use an intervention that was proven to be net beneficial in small and/or short duration trials^c^

^a^London (2020)

^b^London (2019)

^c^Stegenga (2016)

## Section 4: COVID-19 case studies

### Case study 1: Drugs for COVID-19

Early in the Spring of 2020, a vast number of compounds were available to be tested against SARS-CoV-2. Anecdotal reports, non-randomized trials, and laboratory data had been offered as support for various pharmaceutical interventions, including lopinavir/ritonavir (Chu et al. 2004; Yao et al. 2020; Park et al. 2019), hydroxychloroquine/chloroquine (Ferner and Aronson 2020), remdesivir (Holshue et al. 2019; Grein et al. 2020), convalescent plasma (Sahu et al. 2020), vitamin D (Chiodini et al. 2021), tocilizumab (Xu et al. 2020; Luo et al. 2020; Di Giambenedetto et al. 2020), steroids (Arabi et al. 2018), tissue plasminogen activator (Wang et al. 2020) and many other drugs (Lee et al. 2022).

In response to background uncertainty and the potential for near infinite off-label combinations, a few groups began robust clinical trials agendas. The United Kingdom (UK) RECOVERY study (2020), still ongoing, was developed as a pragmatic, multi-arm, adaptive randomized trial, and had arms including several of the above-mentioned drugs. As early as 6 months into the pandemic, this trial produced strong evidence that the inexpensive and widely available steroid dexamethasone could significantly reduce deaths in patients requiring respiratory support (Horby and Landrain 2020). Other agents tested in RECOVERY, such as tocilizumab and baricitinib, also proved successful in some cases of severe COVID-19 (on top of other immunomodulatory treatments), while several others, including hydroxychloroquine and convalescent plasma, failed to show benefit.

Similarly, the World Health Organization (WHO)’s SOLIDARITY RCT (2020) was an adaptive trial that set out to test four experimental therapeutic strategies (remdesivir, lopinavir/ritonavir combined, lopinavir/ritonavir combined with interferon-beta, and hydroxychloroquine or chloroquine) and rapidly alter trial parameters as results emerged. It resulted from an international collaboration which succeeded in producing robust results independent of funding by drug manufacturers. In this trial, all interventions were ineffective for relevant clinical outcomes. For remdesivir equipoise persisted on specific research questions or clinical indications because of conflicting results with evidence from the ACTT-1 trial (Beigel et al. 2020) and there were further attempts to disturb equipoise by repeating trials in different groups (Gottlieb et al. 2021; Ali et al. 2022).

The hydroxychloroquine case is paradigmatic of what can happen when interventions are widely implemented without high-quality evidence of net benefit. In the first months of the pandemic, this antimalarial drug was repurposed for severe COVID-19 after reports of success in early clinical studies. Expected benefits were considered by some to outweigh harms and several countries granted it emergent approval (including the United States [US] Food and Drug Administration [FDA], which then revoked approval in mid-2020). Worldwide shortages for its prior approved indications (e.g., rheumatological conditions) followed. Contrary to popular belief, RECOVERY showed no relevant clinical benefits, and, more worryingly, subsequent studies showed important safety concerns. Likewise, the SOLIDARITY's interim analysis and further investigation revealed similar findings and the FDA eventually revoked its authorization.

Other pooled efforts included the multiplatform, multinational collaboration between the REMAP-CAP, ACTIV-4a and ATTACC adaptive trials (2021). By virtue of their unprecedented cooperation, seeking to maximize speed, minimize competition and enhance external generalizability (Neal et al. 2022), investigators were able to rapidly produce practice-changing evidence. In December 2020 for example, their findings rebutted the widespread belief, based on observational evidence, that antithrombotic strategies in critically ill patients were beneficial. Further randomized trials tried to clarify persisting uncertainty regarding different patient populations and dosing strategies and confirmed an increased risk of bleeding in the critically ill subgroup, suggesting a net harm from the intervention—*contrary to prior observational data* (Wahid and Ortel 2021).

### Summary of case study 1

Research on COVID-19 drugs illustrates several key points. First, there was often significant equipoise regarding a range of research questions before, during and after several pharmaceutical interventions were first approved and/or recommended. Equipoise justified high-quality experimental pharmaceutical research being conducted from the outset of the pandemic. The aim was to try to establish a favorable risk–benefit ratio for drug interventions before widely recommending them.

Second, epistemic standards were generally high for most drug interventions, at least in initial trials. Key drug research initiatives were arguably most successful when they relied on RCTs to generate data. Third, equipoise was often resolved via successful RCTs establishing the net benefits or harms of many drug interventions for specific groups. Fourth, some drug interventions that were implemented in the hope of net benefit but without good evidence were eventually shown to be ineffective and/or produce net harm. When high-quality research data are not available, particularly in PHEs when people feel compelled to act and can easily misjudge expected benefits and harms, the above examples of pharmaceutical research illustrate why it is an ethical obligation to generate robust evidence, and also that high-quality research can continue despite methodological and practical obstacles during PHEs.

### Case study 2: COVID-19 vaccines

Covid-19 vaccine research included notable successes but many missed opportunities and ethical failures. The initial approval of the SARS-CoV-2 vaccines was based on large randomized trials powered for symptomatic COVID-19 (Baden et al. 2020; Voysey et al. 2021; Polack et al. 2020; FDA 2021b). The positive results for several vaccines represent an outstanding scientific achievement. Where things get more problematic is how vaccines were rolled out and different vaccine policies were deployed, and the lack of additional research in populations at low risk of disease (Prasad 2022). For example, when the FDA approved Pfizer’s vaccine in August 2021, it was contingent on Pfizer performing post-marketing safety studies on the incidence of myocarditis (FDA 2021a). This was particularly important given the appearance of worldwide safety signals in young people, yet these studies have not been completed or made public at time of writing (September 2023) (Krug et al. 2022).

### Vaccine implementation

The near-simultaneous approval of different vaccine products with similar efficacy but different dosing schedules gave rise to difficult questions regarding vaccine prioritization (Rid et al. 2021). Overall, the success of initial vaccine doses was confirmed by early real-world data, especially among high-risk and elderly patients. In the face of limited vaccine supply, residual uncertainties (e.g., regarding the effect of vaccines on transmission) and looming high COVID-19 death tolls in early 2021, vaccine strategies were mostly based on ethical and political considerations—resulting in divergent policies at the global level. Evidence for policy was often derived from national epidemiological data and decisions were guided by mathematical models (Tuite et al. 2021). For instance, the UK rolled out vaccines with a clear age-based prioritization strategy and focused on maximal first dose coverage via delayed second doses of the Oxford/AstraZeneca vaccine based on models suggesting that these strategies could prevent more deaths and hospitalizations. Remarkably few countries followed the latter policy, which was later estimated to have averted between 4–9 thousand UK deaths over the first ten months of the campaign (Department of Health and Social Care 2021; Joint Committee on Vaccination and Immunisation 2021; Keeling et al. 2023). This was an early example of divergent policies regarding vaccine dosing strategies, an issue that continued throughout the pandemic.

### Booster vaccination

Until mid-2021 there was high-quality evidence that expected benefits from widespread vaccination in most seronegative adults outweighed the risks. From 2021 onwards, further doses started being recommended or mandated with very little, if any, evidence of global benefit. What was under-appreciated was that baseline conditions had changed significantly. With most people either vaccinated or having been previously infected (or both) and the emergence of milder variants, it became strikingly less likely that there would be an additional individual benefit (i.e., absolute reduction in the risk of severe disease) from subsequent vaccine doses for many if not most members of the general population. Evidence that vaccines halt transmission went from scarce to null, with outbreaks among double-vaccinated individuals recorded from May 2021 (Bengali 2021).

Despite the above, and the majority of COVID-19 outcomes being mild by 2022 (especially for the non-elderly (Pezzullo et al. 2023)), public health agencies faced policy questions regarding third or subsequent vaccine doses. Boosters were approved based on increasingly loose criteria and evidence standards, leading to globally divergent policy recommendations in the absence of evidence (Offit 2023).

In the US, two top FDA advisors resigned in late 2021 in protest against universal boosting. The policy situation has been aggravated as updated boosters (some bivalent) have even less evidence of clinical benefit—with evidence derived from observational studies, non-clinical outcomes (such as the generation of antibodies), or even non-human data (FDA 2022a, 2022b). This represents a significant decline in evidentiary standards compared with the evidence on initial doses at the time of regulatory approval. Nevertheless, booster doses continue to receive emergency use authorization (EUA) by the US FDA, despite concerns expressed by at least one member of the FDA Vaccine Advisory Committee (Offit 2023; New England Journal of Medicine 2023).

### Vaccination of children

In line with the deterioration of epistemic standards for vaccine approval in adults, vaccination of children also raised serious concerns—not only with regards to regulatory pathways, but also the balance of potential benefits and harms. Children’s vaccine trials used antibody levels as primary endpoints, which is arguably inadequate. This is because antibody levels are not in themselves benefits for vaccinated individuals (or for others), and since measuring antibodies requires only small numbers of study participants, such studies are (even more) statistically underpowered for potential harms, an example of wider lack of attention to harms in clinical research (Stegenga 2016; Kraaijeveld et al. 2022). In the absence of randomized trials, experts relied on case–control studies to infer the vaccine’s clinical efficacy in those aged 5 to 11 years old (Price et al. 2022), but these have severe limitations for causal inferences. Case control studies hinge on the idea that cases and controls are drawn from the same population, but children hospitalized with diseases other than COVID-19 (the control utilized by many papers) are a different population (one of vulnerable children or those with underlying medical problems) compared with healthy children. Given the increasing prevalence of previously infected children (in whom the risk of disease upon reinfection is extremely low), vaccine effectiveness would be at best modest. In those 6 months to 4-year-olds, Pfizer was first tasked to conduct a trial evaluating non-clinical endpoints, but even those findings were disappointing. Only later was EUA eventually granted for Pfizer (which modified the protocol to add a third dose) and Moderna. Trials have yet to show clinical efficacy in this age group—and in most countries very few parents (i.e., less than 10%) of young children have chosen to vaccinate (Lopes et al. 2022; Suran 2022).

### Adverse effects of vaccines

Another important factor in the equation of COVID-19 vaccine policymaking is the rare but significant risk of myocarditis with mRNA vaccines, particularly in young males. Some countries in Europe have restricted vaccination in this age group, but the US has not followed. By the second half of 2021, after the first safety signals were already clear, some experts raised questions and demanded better evidence on dosing schedules, target populations and risk–benefit analysis for better deployment of vaccine policies in younger adults (Prasad et al. 2021). Neither the CDC nor FDA have demanded better evidence nor proactively changed their conduct to a more cautious approach. Other countries like Norway loosened recommendations for adolescents (they could choose whether or not to receive just the first dose (Ministry of Health and Care Services 2022)), acknowledging a potential risk of heart damage. This global divergence in policy again suggests equipoise and/or significant disagreement among experts regarding the balance of benefits and harms of mRNA vaccines in young healthy adults and adolescents. Such disagreements are sometimes about value judgements (whether imposing rare risks on young healthy people is acceptable given that other vaccinated individuals may benefit) but also about evidence (whether estimates of both risks and benefits in these age groups are adequate to inform policy recommendations, given the lack of high-quality research addressing these questions).

### Summary of case study 2

This case study illustrates two key points. First, COVID-19 vaccines proved to be another major success amongst trialed interventions, at least in terms of the individual benefits of initial doses in most adults. Vaccines were initially a highly successful intervention precisely because research lived up to high ethical and epistemic standards in a timely manner despite the challenges of a PHE setting. These outcomes were appropriately tested in large RCTs and, in a matter of weeks or a few months, evidence suggested net individual benefit for several experimental vaccine candidates (thus resolving uncertainty and equipoise).

But initial RCTs could even have been improved in two important ways: (1) testing for (asymptomatic) post-vaccination infection to determine the extent to which vaccines blocked infection and transmission (Kahn et al. 2018), and (2) testing for duration of protection over a longer period of follow-up. Both goals would have been achievable—and, arguably, ethical—including via prolongation of placebo arms with young healthy adult participants (Rid et al. 2021).

Second, epistemic and ethical standards deteriorated over time and there was often inadequate research in low-risk groups including young healthy adults, children, and those with immunity from past infection. As of today, we still do not know the optimal number of doses for individuals at different ages, at different risks, and based on prior infection. Much current guidance in the US, but not in Europe, treats people indistinguishably recommends or mandates the same vaccine schedule for widely differing individuals, arguably producing an inferior balance of risks and benefits compared to more nuanced policy options (Bardosh, Krug, et al. 2022).

**Fig. 2:**
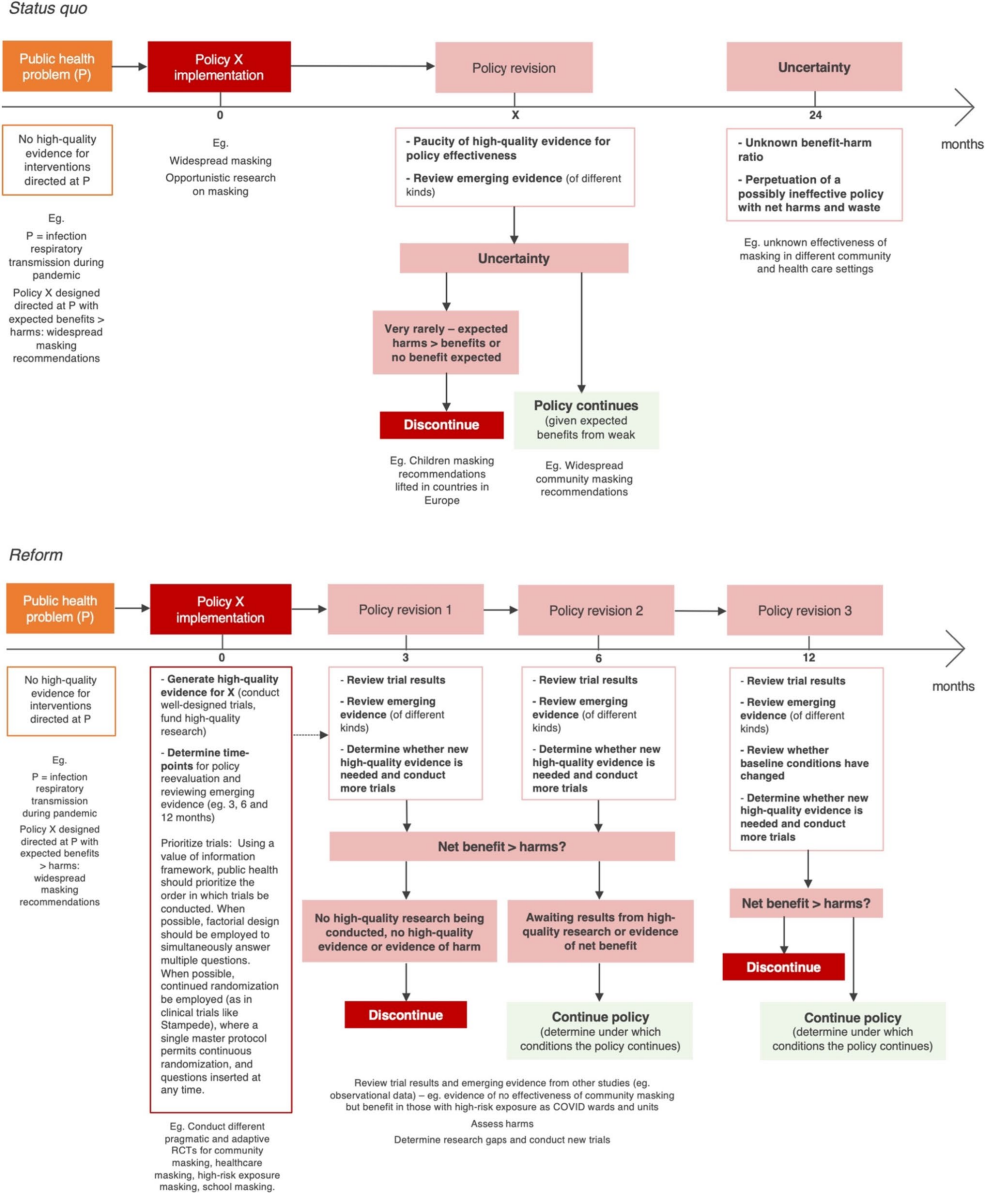
Comparison between proposed public health policymaking reform (a suggestion) and status quo

This situation would have been improved by both (1) larger and more rigorous initial vaccine trials in young and low-risk individuals (including detailed collection of safety data, e.g., regarding mRNA vaccine myocarditis) and (2) encouraging high-quality research, particularly randomized implementation trials designed to assess vaccine rollouts. When assumptions change as was the case with COVID-19 vaccines, an originally beneficial policy can become a net expected harm for millions (Bardosh et al. 2022b). As conditions changed, and most countries had their populations immunized (by vaccination and/or natural infection), there were pragmatic issues to be clarified through similar rigorous techniques and, here, vaccine rollouts did not follow the same scientific standards compared to initial vaccine trials (Fig. 2).

### Case study 3: Non-pharmaceutical interventions

**Fig. 3:**
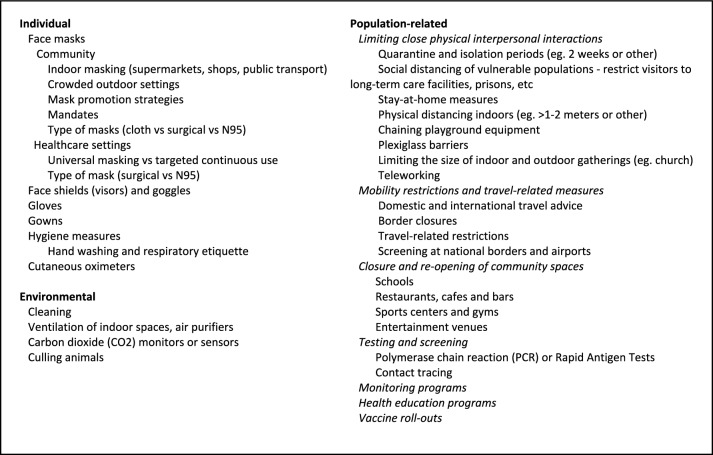
Examples of Non-Pharmaceutical Interventions employed during the COVID-19 pandemic

Contrast the above pharmaceutical case studies with research regarding NPIs (Fig. 3 shows examples of NPIs). When it comes to these interventions, we have close to zero prospective experimental data (Høeg and Prasad 2023) because opportunities to learn about the benefits and harms of NPIs were squandered (McCartney 2020; Bain et al. 2022). Early in the pandemic, policymakers relied mostly on aggregate national or regional data on observed disease incidence associated with different non-pharmaceutical policy responses. Notwithstanding, the majority of (mostly observational) studies of NPIs had a high risk of bias and/or confounding (Talic et al. 2021). Adding to the ethical complexity of policy options, many of these interventions were recommended, mandated, or enforced with varying degrees of vigor.

Consider masking, which became a controversial issue in part because the optimal use of masks remains uncertain. Few randomized trials were run during the pandemic on community mask use, which was previously considered to have little to no effect for influenza (Jefferson et al. 2023). One US government Pandemic Influenza Plan from 2017 does not even include the word “mask” (CDC 2017). Two years and a half into the COVID-19 pandemic, two RCTs were published—one was an individual RCT from Denmark, the other a cluster RCT from Bangladesh (Bundgaard et al. 2021; Abaluck et al. 2022). Neither trial found evidence that masks were highly effective (despite observational data and consensus-based recommendations or mandates from many public health agencies). No mask trial enrolled children. No trial examined the role of masking after vaccination. No trial studied the effect on airplanes or other modes of public transit. The updated Cochrane collaboration concludes, “There is uncertainty about the effects of face masks.…There is a need for large, well-designed RCTs addressing the effectiveness of [such] interventions in multiple settings and populations, as well as the impact of adherence on effectiveness, especially in those most at risk of [acute respiratory infections].” (Jefferson et al. 2023).

In healthcare settings, mask recommendations in 2021–2022 from WHO and US Centers for Disease Control and Prevention (CDC) for staff caring for COVID-19 patients are divergent—WHO recommended medical (surgical) masks, whereas the CDC advocated for N95 respirators (WHO 2020; Loeb et al. 2022). This arguably reflects significant equipoise due to disagreement and/or uncertainty (Fig. 1).^4^ Late 2022 saw the publication of the first RCT on this issue (Loeb et al. 2022). The trial’s results were consistent with there being little to no difference between N95 and medical masks in healthcare workers.

Early in the pandemic, calls for more randomized trials of masks and other NPIs were largely ignored. In September 2020, McCartney published a paper calling for better evidence on non-drug interventions for COVID-19 (McCartney 2020). As she argues, whereas we take drug trials seriously in part because we recognize the possibility of drug-related harm, we cannot presume that non-drug interventions won’t do harm or waste resources. Cristea et al. (2020) comprehensively argued for the need for NPIs to be tested in RCTs, stressing the uncertainty about the magnitude of harms that these measures may induce. Fretheim and colleagues brought particular attention to the need for randomized trials of school closures (Fretheim et al. 2020). About 18 months into the pandemic, urgently needed scientific evidence regarding NPIs was still sparse (Hirt et al. 2022) and a collaboration of global experts aiming to foster high-quality research on Behavioural, Environmental, Social and Systems Interventions to stop COVID-19 made another call for more research (Glasziou et al. 2021).

We concur with those who appealed for high-quality experimental research. Rigorous experimental studies should have replaced observational studies, as the latter have very limited inferential power for the kind of causal inferences on which policymakers should arguably rely. Indeed, study designs that involved controls (e.g., regression discontinuity studies) and/or pseudo-randomization (e.g., natural experiments) consistently showed that, for example, school masking (Coma et al. 2023), curfews (de Haas et al. 2022), and hard lockdowns were less effective than in some observational studies (Butterworth et al. 2022; Jamrozik 2022), and demonstrated that longer durations of school closures were associated with increased mental health harms (Felfe et al. 2023).

Very few NPI trials were conducted and NPI policy was therefore often based on low quality observational data. Once policies were in place (and sometimes mandated), randomized trials were often deemed unnecessary or unethical, sometimes because of the view that equipoise had been disturbed (despite a lack of new high-quality data) (C-SPAN 2023). However, this approach underestimated residual uncertainty and undermined the ethical standards of research for public health policy. In sum, NPIs should have been tested in high quality experimental designs such as RCTs - including in cases where many experts believed (on a fragile epistemic basis) that expected benefits outweighed harms. Below, we consider and respond to several prominent objections regarding the feasibility, utility, and ethical acceptability of RCTs in PHEs.

## Section 5: Objections and responses

### Objection 1: The precautionary principle

The precautionary principle (PP) was upheld several times during the recent pandemic with regards to NPIs. Briefly, on some interpretations of PP, it is acceptable for decision-makers to adopt and legitimize restrictive measures when "scientific information is insufficient, inconclusive or uncertain” (Comission of the European Communities 2000, 7) and risks to human health are considerable. It was widely used as a justification for restrictive public health measures and mandates, particularly in the very beginning of the pandemic. The invocation of PP indicated a situation of uncertainty—i.e., that most implemented policies were introduced on a basis of little or no evidence and/or evidence of little or no benefit. In April 2020, for example, Greenhalgh et al. (2020) argued that it should be applied to masking policies.

Since there was uncertainty about both the virus and the interventions, it was (on this account of PP) better to introduce NPIs as precautionary measures, and some might think that the mere possibility of benefit may render research unnecessary or unethical. On the contrary, uncertainty strengthens, rather than undermines, the case for research regarding implemented measures. First, uncertainty is typically associated with significant equipoise and in any case may itself justify science aiming to resolve uncertainty. Second, uncertainty at the outset of a PHE entails that interventions might have a range of benefits (including no significant benefits) and/or a range of harms (including no significant harms) and costs. Since these potential benefits, harms, and costs have major implications for society, there will often be immense social value associated with research on interventions for PHEs. Third, since key principles of public health ethics include both the “Need for Evidence” and “the Least Restrictive Alternative”, there is arguably an even stronger ethical rationale to provide rigorous evidence (via research) for interventions that are not merely recommended but *mandated*, as was the case for many NPIs (Jamrozik 2022). Overall, while PP and/or uncertainty might justify action at the start of a pandemic, as time goes on there should be an expectation that policy is justified by increasing epistemic certainty regarding the net benefits of interventions, and this certainty is best supported by high-quality science.

### Objection 2: Interventions are like parachutes

During the pandemic, it was sometimes claimed that certain interventions were akin to "parachutes", particularly NPIs such as masking (Greenhalgh et al. 2020). A classic expression of this idea is the British Medical Journal (Smith and Pell 2003) article that satirically noted the absence of randomized trials for gravitational challenge. In other words, we don’t have trials proving that wearing a parachute when falling from an airplane is lifesaving, but the net benefits of using them are clear from non-randomized evidence. This might be considered a strong ethical rationale for the implementation of some policies without requiring high-quality experimental evidence of net benefit.

Several teams have pointed out the limits of the parachute analogy. First, appeals to the parachute analogy often refer to practices that have in fact been tested in a randomized fashion, undermining the analogy and its conclusion (Hayes et al. 2018; Xu and Prasad 2022). Second, few medical practices have effect sizes of the magnitude of parachutes (Glasziou et al. 2007). In an analysis by Pereira and colleagues (Pereira et al. 2012), the authors note only one practice in the entire Cochrane database with a very large effect on survival (extracorporeal oxygenation for neonates, with a roughly 40% reduction in all-cause mortality (still far lower than the presumed ~99.9% mortality reduction for parachutes) (Mugford, Elbourne, and Field 2008). It appears likely that most if not all NPIs are (1) not as effective as parachutes, and (2) amenable to randomized trials.

### Objection 3: Disturbance of equipoise or change in expert consensus

In January 2022, CDC director Rochelle Walensky asserted that "any mask is better than no masks at all" (The White House 2022). In response to questions regarding the lack of mask RCTs during the pandemic, Walensky stated: "I’m not sure anybody would have proposed a clinical trial because, in fact, there wasn’t equipoise to the question anymore." (C-SPAN 2023). Of note, although there were no mask trials in the US, such trials were conducted in Denmark, Bangladesh, and (though published later) Guinea-Bissau (Bundgaard et al. 2021; Abaluck et al. 2022; Nanque et al. 2023).

Simplistic views of equipoise such as those expressed by Walensky are problematic for several reasons. First, while many experts changed their views on masks in early 2020 (from a prior view that in community use masks have little to no benefit against respiratory viruses to a view that masks are likely to be highly beneficial in community use), there was only a fragile epistemic basis for this change. For example, a vocal group of scientists self-identifying as mask advocates “realized” in early 2020 that existing data supported the use of cloth masks (contra widespread pre-pandemic consensus regarding cloth masks) without there being any new high-quality evidence to justify this change.^5^ Since then, no new high-quality evidence has been produced to support a large beneficial effect of community masking and few public health agencies recommend the use of cloth masks, suggesting that they are now widely believed to be ineffective. Further evidence on other types of masks has also been consistent with small or no benefit (Jefferson et al. 2023). This illustrates that the apparent disturbance of equipoise by a change in expert views without strong evidence is not the same as a change in views based on strong evidence.

Second, there was arguably an element of “curated consensus” via political or peer pressure and/or fears about a novel virus rather than via evidence-based persuasion. Masks were seen not only as a (potentially) effective intervention, but also perceived by healthcare workers and members of the public as “talismans” that “increase…*perceived sense of safety*, well-being, and trust [italics added]”, as expressed in a top medical journal (Klompas et al. 2020). Evidence-based pandemic interventions typically aim to measure endpoints such as a reduction in transmission or disease (rather than *perceived* safety), but efforts to measure these outcomes might have been seen as a threat to the *perceived* benefits of masks.

Third, simplistic accounts of equipoise cannot account for the wide disagreements between different experts or expert groups, such as the CDC and WHO. These disagreements, and widely divergent national mask policies, occurred in the face of the same evidence. Such disagreements, and uncertainty about which interventions work best, are precisely what should drive high-quality research, which commits to higher epistemic standards and has greater potential to resolve equipoise on stronger epistemic grounds. In the case of masking, inquiring whether masks work should be further broken down into different research questions (depending on the setting, study population, etc.) where trials might shift collective expert views. As shown above, there are several trial designs that can feasibly and pragmatically help approach those questions. Appealing to high-quality research to generate evidence of effectiveness (or net benefits) is our best attempt at resolving equipoise and determining what the most cost-effective use of interventions might be.

### Objection 4: Layered interventions or the “Swiss cheese” approach to public health

It has been claimed that multiple interventions for COVID-19 need to be implemented in “layers” in order to protect individuals, and this claim is often represented by the Swiss cheese metaphor (Escandón et al. 2021). The naturally perforated “cheese” slices, or imperfect layers of interventions, consist of, for example, physical distancing measures, masks, hand hygiene, eye protection, ventilation, testing strategies, and vaccines, among others. It has been claimed that the complexity of such multi-intervention strategies presents a challenge for the feasibility and/or ethical acceptability of RCTs (Escandón et al. 2021).

These apparent concerns do not undermine the case for rigorous trials of interventions. Randomized trials (including adaptive platform trials) are ideal research designs for evaluating combinations of interventions and determining whether the *addition* of one specific intervention produces net marginal benefits or harms. For example, cancer trials routinely test combinations of chemotherapies in different combinations. This allows for assessments of combinations of interventions and, specifically, whether *additional* interventions (or changes in the way interventions are delivered) improves or worsens outcomes. In other words, rigorous trials are a method to evaluate the hypothesis or assumption (inherent to the Swiss cheese model) that more interventions are always better than fewer interventions.

Of course, trials of some public health interventions are in some respects more complex than randomized trials of interventions for individual patients such as chemotherapy. One reason is that some public health interventions, especially for infectious diseases, may have indirect effects—the classic example being that vaccines often have some effect on the transmission of infection to others. Yet it is feasible to design trials of public health interventions to measure these effects (Wolfenden et al. 2021; Digitale et al. 2021). A second reason is that population level outcomes (size of epidemic, number of hospitalizations over time, etc.) are affected by many additional factors in addition to specific interventions. However, these factors can be reasonably controlled for in cluster-randomized trials, for example, by using a combination of interventions in one (sub-)population and the same combination *plus or minus one intervention* in a similar but separate (sub-)population.

A final possibility is that the Swiss cheese model in the context of infectious diseases implies that each individual intervention has at most weak effectiveness against (acquisition and/or transmission of) infection. It is true that rigorous studies, whether experimental or observational, require large sample sizes or long periods of follow up to detect *small effects*. This might present a practical challenge to studying the effectiveness of each individual “layer” of interventions. However, insofar as (different) combinations of interventions produce larger effects, these should be readily detectable in rigorous trial designs. While it might be difficult for trials to *rule out* very small effects of individual interventions, one might also question whether interventions with no detectable effects are likely to be cost-effective at the population level.

### Objection 5: Urgency or insufficient time for rigorous trials to produce useful results

A final objection is that RCTs produce results too slowly to be useful for policy formation during an evolving PHE (Brouqui and Raoult 2020; Adebamowo et al. 2014; Tufekci 2020). Many people might believe that desperate times require emergency use of interventions without waiting for the results of research (London and Kimmelman 2020). For this reason, the 2002 Council for International Organizations of Medical Sciences (CIOMS) research ethics guidelines note:

When facing a serious, life-threatening infection, many people are willing to assume high risks and use unproven agents within or outside of clinical trials. However […] many promising experimental agents may not be safe and effective, and experimental interventions must be systematically evaluated in clinical trials. […] Widespread emergency use [of unproven interventions] with inadequate data collection about patient outcomes must therefore be avoided. (2016, 77).

We agree with this statement, and our response to the urgency objection is threefold. First, there is an (at least partly) ethical case in favor of controlled research designs during PHEs, precisely because experimental interventions may not turn out to be safe, effective, or cost-effective. Further, studies *comparing* interventions can help policymakers select the best interventions. Second, coronavirus vaccine RCTs produced results within (in some cases) less than two months and resulted in major rapid changes to policy, even allowing for the additional time required to manufacture and distribute vaccines. Many NPIs, for example, do not even require this additional manufacturing time and those that involve rules and regulations can be changed rapidly during a PHE. Producing high-quality data allows such changes to be based on evidence rather than more arbitrary factors (Bain et al. 2022). Third, additional capacity building to prepare trial designs and infrastructure prior to future PHEs would permit the launch of trials of many interventions soon after the beginning of a crisis and produce results within a few months showing either that policies were associated with significant effects (i.e., net benefits or harms) or not. Policies associated with no effects or net harms should arguably be revised.

## Section 6: Time for change: a proposal for public health reform

In this section we propose a reform for the next PHE that would satisfy the need for (1) rapid policy implementation, (2) high-quality evidence generation, (3) sustainable and ethical longer-term policy responses, and (4) accountability. If public health practice is to live up to high ethical and epistemic standards, we contend that policy should be based on sound evidence wherever possible. This means that whenever a non-trivial policy is not grounded in high-quality evidence that expected benefits outweigh harms, there should be a planned approach to conduct high-quality research to collect such evidence. We intend our proposal to apply to all key public health policies in PHEs, i.e., those that apply to many people, involve significant (public) costs, and/or have large potential benefits or harms.

Given the need for rapid policy implementation, there should be a plan to (1) begin well-designed trials early and (2) review emerging evidence at preset time points. At these time points, public health authorities should (3) decide whether the policy continues or is suspended. Our suggestion is that, by default, policies should be reviewed at 3, 6 and 12 months after the initial policy implementation. Figure 2 depicts the *status quo* versus our proposed framework.

This stepwise approach has several advantages. First and foremost, it is rooted in high ethical and epistemic standards of health research allowing, in principle, epistemic progress. It permits selection of net beneficial interventions and cessation of non-beneficial or harmful interventions. Drugs and vaccines for COVID-19 were initially quite successful policies as judged by these new standards. For most widely recommended drugs (e.g., dexamethasone) as well as the first vaccine rollouts, high-quality randomized trials were rapidly deployed before implementing those policies and provided results within several months. But trials should also continue where background conditions change or where there is reason to think that specific groups face different benefits or harms—for example, further high-quality trials of vaccines could have supported policy revision for young healthy people and/or those with immunity within 6 or 12 months; further trials of anti-viral drugs in highly immune populations would help to determine their current cost-effectiveness.

Second, our proposal allows for policies to be implemented on weaker evidence bases, insofar as there is a plan to generate and collect evidence during or after policy implementation. This is consistent with invocations of the precautionary principle at the outset of a PHE and could have happened for most NPIs including by being facilitated by coordinated networks of research. Such networks were assembled for research of pharmaceutical interventions and proved to be very successful (eg. multiplatform trials). Beyond research institutions themselves, appropriate policies for coordination and/or data linkage across multiple government agencies would help to facilitate trials of some NPIs and capacity building in this area would be particularly valuable prior to the next PHE (Kinyanjui et al. 2020).

Third, our proposal is also a more efficient and accountable approach to policymaking. Preset timepoints guarantee that policymakers pause to review emerging evidence and consider ceasing ineffective or even harmful policies, thereby redirecting resources to more effective or beneficial ones. Health agencies can be held accountable for failure to review available data as planned, unless there is good reason for delay.

This pragmatic framework arguably secures higher ethical and epistemic standards than the *status quo*. Rigorously designed standard or pragmatic RCTs would become the rule rather than the exception in PHEs. This is because uncertainty is generally the rule at the outset of PHEs (and in medicine and public health more generally). Reducing uncertainty and disturbing equipoise via high quality evidence should be the goal, especially during emergencies, and should be applied to both pharmaceutical and non-pharmaceutical interventions.

## Conclusions

In this essay we argue that high-quality research is ethically obligatory before, during, and after implementing public health policies, including during emergencies. We defend that equipoise is an appropriate ethical standard for conducting both clinical and public health research. Claims that research was unnecessary or unethical during the recent pandemic appeared to rely on over-optimistic assumptions that the expected benefits of selected interventions would outweigh harms on a population level (thus risk to participants was unacceptable or unnecessary). However, uncertainty and (global) equipoise were the norm. Among other things, this was reflected in a wide variation in policy choices at the international level. Our analysis of the strategies employed during the pandemic—drugs, vaccination, and NPIs—suggests that public health agencies should often have demanded better evidence before, during and after policy implementation, especially for NPIs.

Large randomized trials of drugs and vaccines were a success—there was generally a better understanding of the ethical rationale for randomized trials and their epistemic superiority in the context of healthcare interventions, particularly after several reversals (drugs that turned out to be less effective than initially believed). But where non-pharmaceutical interventions are concerned there has been a widespread misunderstanding that equipoise was disturbed by the implementation of policy (or its endorsement by some public health experts), even where this implementation was not based on strong evidence. Despite several appeals, high-quality NPI research was very rarely carried out. Further, there should be more discussion of the importance of randomized trials of policy in academic research ethics (MacKay 2023; Bain et al. 2022; Høeg and Prasad 2023).

We do not wish to reduce complex moral situations of decision-making, particularly those involving judgments of acceptability of risk, to a mere matter of doing more RCTs. Our main point is that expert judgements about the merits of interventions must be guided by high-quality data in addition to appropriate ethical and procedural values. In many cases of expert disagreement, the dispute is about the evidential support (or lack thereof) for the benefits and/or harms of a given intervention. High-quality research is the most reliable way of improving estimates of these outcomes, and moral or policy debates built on inaccurate estimates may have serious consequences. In some cases, policy disputes are instead (or also) about moral questions, such as whether (or the conditions under which) it is ethically acceptable to impose a low probability of serious harms via vaccination on young healthy individuals. Some of our arguments nevertheless apply insofar as such disputes would be better informed by rigorous data collection (even if this is in non-randomized studies)—as demonstrated by the failure to collect rigorous data on the risks of mRNA vaccines prior to mandating them in individuals among whom these vaccine-associated risks turned out to be highest.

We advanced a proposal to reform public health policymaking. Agencies making recommendations should be responsible for planning a stepwise approach to generate and collect robust experimental evidence before, during and/or after policy implementation. We suggested this should be done with preset timepoints (eg. at 3, 6 and 12 months) to review accruing evidence and reevaluate policies. This pragmatic research framework could help to hold policy to high ethical and epistemic standards, thereby improving accountability. The alternative—aligning scientific expert opinion with political imperatives and/or expert consensus without evidence—risks imposing ineffective or harmful interventions on whole populations as well as premature foreclosure of important scientific questions regarding the benefits and harms of policy decisions. In the long term, this foreclosure may undermine trust in science and public health.

## Footnotes

1 NPIs are here defined as interventions which do not contain pharmaceutical substances aimed at preventing and/or controlling a disease or condition.

2 Well-designed observational studies may also provide useful information regarding effectiveness, and sometimes more useful than a poorly designed randomized controlled trial (RCT). We focus on RCTs because there is reason to think that well-designed RCTs are often more reliable than well-designed observational studies and we later advance a policy proposal for the implementation of RCTs in particular.

3 “Policy” is here considered in a broad sense, including clinical recommendations and public health measures.

4 While WHO makes global recommendations that include low-income countries, there are also divergent guidelines in high income countries (i.e., some jurisdictions recommend medical masks, others recommend N95s, etc.) (Oltermann 2021). Thus, these divergent recommendations cannot be entirely explained by cost or other pragmatic considerations in low-income settings.

5 See “Masks4all” (Howard et al. 2020).
